# The Effect of Socioeconomic Factors on Maternal Perinatal Depressive Symptoms and the Contribution of Group Prenatal Support as a Preventive Measure

**DOI:** 10.1089/whr.2022.0042

**Published:** 2023-01-24

**Authors:** Konstantina Natsiou, Sokratis E. Karaoulanis, Konstantinos Dafopoulos, Antonis Garas, Konstantinos Bonotis

**Affiliations:** ^1^ Midwife, Psychoprophylaxis Department, Health Center of Larissa, Larissa, Greece.; ^2^ Department of Psychiatry, University Hospital of Larissa, University of Thessaly, Larissa, Greece.; ^3^ Department of Obstetrics & Gynecology, Faculty of Medicine, School of Health Science, University of Thessaly, Larissa, Greece.

**Keywords:** psychoprophylaxis, maternal depression, maternity blues, life stress events, pregnant women, postpartum period, economic crisis

## Abstract

**Background:**

The European and Greek financial turmoil that began in 2007 has had adverse health consequences. Stillbirth, low birth weight, infant mortality, and maternal suicide have all increased. The purpose of this study was to evaluate whether socioeconomic factors contribute to postpartum blues, and whether psychoprophylaxis with group prenatal education and support may have a beneficial effect.

**Materials and Methods:**

The sample study comprised 414 pregnant women equally divided into psychoprophylaxis or standard care. There were six psychoprophylaxis sessions, with two each week lasting 2 hours each in groups of five people at the urban health center of Larissa, Greece. A questionnaire was used for data collection, including (1) closed-type questions about sociodemographic characteristics, and medical and obstetric history; (2) the Hamilton Depression Scale; (3) a Blues Questionnaire; (4) the Holmes and Rahe stressful life events scale; and (5) a scale of effects of the economic crisis. Differences between the two groups and within the groups at different time points were assessed by two-way repeated measures ANOVA tests.

**Results:**

Maternity blues scores, depression scores at all time points, life stress event score, and financial difficulty score were all significantly related to each other in both groups at all time points (*p* < 0.01). The correlation between financial difficulties and depression/maternity blues disappeared after delivery in the intervention group. Financial difficulties, depression, and psychoprophylaxis sessions emerged as independent prognostic factors of maternity blues score, the group variable being most significantly associated with maternal blues.

**Conclusion:**

Although financial status as well as depression continued to play a role, the deterrent contribution of psychoprophylaxis was the most important parameter in the final maternity blues prognostic model. The results of our study show a potential for prevention and suggest interesting hypotheses for future interventions.

## Introduction

The economic crisis that broke out in Europe and America in 2007 spread throughout the world and affected all economy sectors to a great extent.^1^ Cuts in health sector funding and an increase in stressful events and depression resulted in severe health effects.^1^ A major consequence of the economic crisis was the increase of all causes of mortality, while another one was the increase of depression and anxiety symptoms, mainly due to the increased unemployment, and the increase of alcohol consumption, smoking, and drug use.^2^

Greece in particular has been hit very hard by the international financial turmoil, which practically lasts till today (typically, the Eurogroup's approval for Greece's exit from the status of enhanced surveillance was given on June 2022; https://www.businessdaily.gr/english-edition/65470_green-light-greeces-exit-enhanced-surveillance-status). Greece was the country with the largest cuts of around 28% compared to <10% for the remaining countries, per capita expenditure on health.^3^ Economic crisis was associated with considerable adverse effects on perinatal outcomes and infant mortality.

The study by Kentikelenis et al^4^ showed changes in four indicators of national health: stillbirth, low birth weight, infant mortality, and suicide. According to data from the Hellenic Statistical Authority, the prevalence of stillbirths increased by an average of 19% during the crisis (2009–2012), compared to the precrisis period (2005–2008). In contrast, the increase in the prevalence of low birth weight during the years 2008–2010 was the continuation of an increase of three decades (79% in total during 1982–2010) and is mainly due to the simultaneous increase of premature births during those years.

Economic crisis unemployment plays an important role in the development of anxiety and depressive disorders and is closely related to suicide attempts. A gradual and steady increase in 1-month prevalence of major depression during financial turmoil was reported, from 3.3% in 2008, to 6.8% in 2009, 8.2% in 2011, and 12.3% in 2013. Data, especially, on maternal mental health are scarce. In a study published in 2022, 15.5% of pregnant women in Greece were at an increased risk of developing depression symptoms; low household income and unpleasant event during pregnancy were related to depression symptoms. It is estimated, however, that about 10% to 15% of new mothers experience depression symptoms. This prevalence justifies the prevention of this global public health problem, especially in women with low socioeconomic status, of whom almost one in four develops postpartum depression.^5,6^

The Lamaze method of psychoprophylaxis is a form of group prenatal education and support. The term psychoprophylaxis is derived from the Greek “psyche,” for soul/spirit/intelligence, and the word prophylaxis. It includes knowledge and practices to achieve a birth without fear and with the least possible pain. With this method, the pregnant woman, using her intelligence, is trained to apply her knowledge at a given moment, to protect her body from pain and her soul from possible injuries. Psychoprophylaxis is considered a method of psychophysical preparation because it concerns the proper preparation of the woman, taking care of her physical and emotional component.^7^

Psychoprophylaxis tools are the theoretical information of the pregnant woman and her partner on issues related to pregnancy, childbirth, and breastfeeding, along with the practice of breathing exercises, relaxation techniques, and self-concentration.^7,8^ Relaxation techniques help the woman to relax, control her body, maintain reserves, and deal with pain. By applying proper breathing techniques at each stage of labor, the patient remains calm, her mind is distracted from the pain, the secretion of endorphins is activated, the pain threshold increases, and the stimulus of pain is partially blocked, thus reducing pain during contractions.^9,10^

The midwife has a duty to explain, to empathize, to cooperate, and to communicate with couples and especially women, to dispel fears and prejudices, and to answer their questions about pregnancy and childbirth, in an atmosphere of free discussion and exchange of ideas and feelings. The international literature has proven the usefulness and reliability of psychoprophylactic preparation for childbirth, while there is no study that considers psychoprophylactic harm.^9,10^ However, its evidence-based evaluation is rare. Reviews of the use of alternative methods of analgesia in childbirth are rare and are characterized by methodological errors. The effect of psychoprophylaxis on the outcome of childbirth has not been thoroughly studied.

In one study,^11^ it was found that the education of the pregnant woman had no effect on the experience of childbirth, the outcome, or the administration of epidural anesthesia or even parental stress. However, there is a lower risk of cesarean section, as it was found in one of the first studies of its kind in pregnant women who used the Lamaze method.^9^ This finding was confirmed in a study in Greece, along with a positive effect on breastfeeding, while a better Apgar at the first minute of life was observed in newborns when mothers had received obstetric psychoprophylaxis, in comparison with those who had not.

Important parameters influencing a woman's mood during the early period of childbirth are her marital and socioeconomic status, any stressful life event, emotional disorders during pregnancy, and a history of depression, as well as complications during pregnancy and childbirth.^12^ Whether psychoprophylaxis can protect against the effects of these factors has not been adequately studied. In their study,^11^ it was found that psychoprophylaxis was no better than conventional methods of preparing for childbirth in reducing parental stress or overall childbirth experience. However, the study was conducted in Sweden in 2009, before the financial crisis and in a health system organized differently from the Greek one. Other studies have focused on specific psychological interventions, outside the context of obstetrics.^4,13,14^

The purpose of this study was to evaluate the contribution of psychoprophylaxis to the experience of childbirth, as well as to the mental mood of the mother, both during pregnancy and during early neonatal period, in the midst of financial crisis, comparing mothers who had attended the program with mothers who had not. The possible contribution of the financial crisis to postpartum blues was also examined.

## Materials and Methods

The study population consisted of 414 women who were monitored in the two hospitals of the city and in private gynecological clinics. The women were completely healthy, caring a single fetus. Some of them had undergone assisted reproduction techniques. The information about the sessions was made by distributing leaflets in private clinics and in two hospitals of the city. There was also a relevant online advertisement. The study took place at the Urban Health Center of Larissa, in the center of the city. After a telephone communication with the midwives of the urban health center of Larissa, a meeting day and time was set for the first lesson. This was a nonrandomized clinical trial (quasi-experimental) study with a control group.

The women were divided into two groups, depending on their desire, those who would attend psychoprophylaxis classes and those who would follow the usual obstetric and gynecological instructions during pregnancy; the standard midwifery care included information on pregnancy stages, possible complications and their early recognition and treatment, possible mood fluctuations, and breastfeeding practices. The women in both groups agreed to participate in the data collection and monitoring and informed consent was requested and given by all women (written consent for the participation and processing of personal-medical data, none refused participation).

The intervention group received psychoprophylaxis according to *Lamaze* method. All 414 women included in the study had no comorbidities; they were capable of speaking and writing Greek fluently and no one had ever received psychoprophylaxis lessons in the past.

The sessions were six in number, took place two each week and lasted 2 hours each (total program duration: 3 weeks) in groups of five people.

First lesson: acquaintance. The objectives of the research were presented and the women completed the first questionnaire. Also, the anatomy and physiology of the female genital system were presented and advice was given to deal with the side effects and possible complications of pregnancy.

Second lesson: childbirth (part 1): Presentation of stages of childbirth and analysis of each stage.

Third lesson: childbirth: (part 2): relaxation breaths for each stage of childbirth, pelvic floor exercises, and stimulation of the muscular system of the pregnant woman; and relaxing music and relaxing breaths at the end of the lesson with low lighting and lying on mattresses.

Fourth lesson: Caesarean section; maternity hospital bag preparation. Repeating relaxation breaths.

Fifth lesson. Breastfeeding: in the breastfeeding lesson, we refer to the benefits of breastfeeding for the newborn and the mother, as well as the composition of breast milk. Reference is made to the mechanism of milk production and how important the benefits of colostrum are. We talk about breastfeeding techniques and breastfeeding problems, for example, mastitis.

Sixth lesson. Neonatal care: we refer to the care of the newborn and possible illnesses, lactation, and postpartum exercise with video projection; other issues are maternity blues and contraception during breastfeeding.

The completion of questionnaires (except for the maternity blues) took place for the first time for both groups at the beginning of the psycho- preparation sessions. Only women in the intervention group after the sixth course completed the questionnaires for second time; the third time was within 10 days proximal to the expected date of delivery (both groups); the women filled out questionnaires for the fourth time within the first 10 days after delivery (both groups). The intervention group therefore completed the questionnaires four times (three before and one after delivery), while the control group three times.

The participation of women was voluntary and women were informed that the data collection serves only research purposes.

A questionnaire was used as a study tool, which included the following:

(A) A questionnaire with closed type (multiple choice, dichotomous, and multithematic question) in relation to the sociodemographic characteristics of the sample and the medical and obstetric history in particular. The design of the introductory questionnaire and its questions was based on valid questionnaires, such as that of the Greek Institute of Child Health.
(B) The Hamilton Depression Scale: The Hamilton Depression Scale was first published by Max Hamilton in 1960 and was designed to measure the severity of depression in already diagnosed patients with major depressive disorder. It includes 23 entries rated from 0 to 2, from 0 to 3, or from 0 to 4, thus giving an overall score ranging from 0 to 63. From subsequent correlations with clinical assessments, the following criteria for the severity of depression off points: 0–7 no depression, 8–16 mild, 17–23 moderate, and ≥24 severe. The time required to complete the scale is 15–20 minutes.^15^ The instrument was used by an experienced clinician and includes questions regarding cognitive and emotional symptoms, helping depression diagnosis in pregnancy. Of note, both clinician-rated and self-rated scales can be effective tools in identifying perinatal episodes of major depression.
(C) Blues questionnaire (BQ): this research tool was developed by Kennerley and Gath to detect and measure postpartum melancholy. The above tool is a valid self-assessment scale, consisting of 28 questions about the emotional state of the placenta. The available answers are “yes” or “no”; therefore, the maximum score is 28 and the minimum is 0. The calculation of the dividing line for the detection of severe melancholy of childbirth (maternity blues) is obtained from the average maximum score of all scars of each study.
According to Kennerley and Gath, the 28 BQ questions can be categorized into 7 symptom groups: primary melancholy, anxiety, hypersensitivity, depression, discouragement, slowing down, and low self-esteem. If the placenta responds positively to more than half of the components of one of the seven symptom groups mentioned above, then it is considered positive for that group. The authors also mention in their article that the group of primary melancholy includes seven symptoms, which are the most common and characteristic of postpartum melancholy. These symptoms are as follows: “I cry easily,” “I feel tired,” “I am anxious,” “I am very emotional,” “My mood is changeable,” “My mood is low,” and “I forget, I am in confusion.”^16,17^
(D) The scale of effects of the economic crisis: to measure the financial situation and ability of individuals, a new scale was developed consisting of 10 questions, each of which is evaluated on a 5-point scale (not at all [1] to very good [5]). The scale examines the individual's ability to meet needs such as “Supply basic daily items” or “Save.”
(E) The stressful life event scale of Holmes and Rahe, which consists of 43 questions of different weight that refer to important life events (marriage, divorce, illness, robbery, etc.). The sum of the individual points leads to an overall score indicative of the psycho-stressful situation experienced by the individual.^18^

The study was conducted according to the declaration of Helsinki—ethical principles for medical research involving human subjects. It was approved by the local ethics committee of the Larissa health center (no. of approval 4210/27-07-18) and by the institutional board (ethics committee) of the Larissa university school of medicine (no. of approval 5796/18-12-19).

### Statistical analysis

Descriptive and inferential statistical analysis was performed. The differences between the two groups (between and within the groups at different time points) were assessed by the two-way repeated measures ANOVA test. The sample size was calculated after power analysis for repeated measures (*a* = 0.05, 1 − *β* = 0.95, effect size 0.10). Correlations were performed with the Pearson test. Measurements exhibited excellent intraclass correlation: 0.910 (0.889–0.918). The level of statistical significance was set at *p* = 0.05. This was followed by multivariate analysis using dummy variables introduced in a linear regression model, after testing for multicollinearity. The statistical package SPSS 22.0 was used.

## Results

Mean age in the intervention and control group was 31.67 ± 5.03 and 29.68 ± 6.41 years old, respectively. Most women were married in both groups. The majority in the intervention group were university graduates (58.0%) versus 39% in the control group. Caesarean section reached 72% in the control group and 64.7% in the intervention group. Percentages for assisted reproduction were 8.2% and 7.2% for control and intervention group. Almost all women in the control group were primiparous, while 72.9% were primiparous in the intervention group (Table 1).

**Table 1.** Demographic and Basic Obstetrics Characteristics of the Two Study Groups

Variables	Group	
	Control (*N* = 207)	Intervention (*N* = 207)
	Mean ± standard deviation	Mean ± standard deviation
Age	29.68 ± 6.41	31.67 ± 5.03
	*N* (%)	*N* (%)
Married	193 (93.2%)	198 (95.6%)
Educational level		
Elementary and Junior high school	23(11.1%)	8 (3.9%)
High school	51 (24.6%)	28 (13.5%)
University	81 (39.1%)	120 (58.0%)
Else (non defined)	52 (25.1%)	51 (24.6%)
Number of labors (primiparous)	206 (99.5%)	151 (72.9%)
Caesarean labor	149 (72.0%)	134 (64.7%)
Assisted reproduction	17 (8.2%)	15 (7.2%)

At baseline, women in the two groups exhibited a statistical difference regarding Hamilton depression scale score (4.31 ± 2.36 vs. 5.09 ± 1.95 for intervention and control group, respectively, *p* = 0.001). A next measurement was reserved for intervention group only, immediately after the psychoprophylaxis sessions (score: 4.20 ± 2.83, phase 2), data not shown. Both groups were then examined at two consecutive time points (phase 3 and phase 4, the latter after delivery). Differences were significantly widened as shown in Table 2 and graph 1, the values in the intervention group continuously declining. Repeated measures analysis revealed significant differences between the various time points in the two groups and within intervention group (*F* = 44.019, *p* < 0.001).

**Table 2.** Differences Between Control and Intervention Groups Regarding Hamilton and Maternity Blues Questionnaire Scores

	Group	N	Mean	Standard deviation	p
Hamilton score-phase 1	Intervention	207	4.31	2.36	<0.001
	Control	207	5.09	1.95	
Hamilton score-phase 3	Intervention	207	4.18	2.65	<0.001
	Control	207	7.75	3.83	
Hamilton score-phase 4	Intervention	207	3.28	2.76	<0.001
	Control	207	8.33	4.80	
Maternity blues score	Intervention	207	7.85	1.70	<0.001
	Control	207	10.61	3.38	

Maternity blues score, depression score at all time points, life stress event score, and financial difficulty score were all significantly related to each other in the control group (*p* < 0.001). However, regarding intervention group, the correlation between financial difficulties and depression/maternity blues was disappeared at phase 4 (after delivery) (Table 3).

**Table 3.** Depression Life Stress Events and Financial Restraints Correlations

			Hamilton score-phase 1	Hamilton score-phase 3	Hamilton score-phase 4	Financial difficulties	Life stress events
Control group (*N* = 207)	Hamilton score-phase 1	*r*	0.600	0.408	0.303	0.335	0.347
		*p*	<0.001	<0.001	<0.001	<0.001	0.001
	Hamilton score-phase 3	*r*		0.737	0.487	0.295	0.269
		*p*		<0.001	<0.001	<0.001	<0.001
	Hamilton score-phase 4	*r*			0.548	0.227	0.230
		*p*			<0.001	0.001	0.001
	Maternity blues score	*r*				0.314	0.267
		*p*				<0.001	<0.001
	Financial difficulties	*r*					0.732
		*p*					<0.001
Intervention group (*N* = 207)	Hamilton score-phase 1	*r*	0.568	0.754	0.183	0.331	0.265
		*p*	<0.001	<0.001	0.008	<0.001	<0.001
	Hamilton score-phase 3	*r*		0.775	0.387	0.220	0.286
		*p*		<0.001	<0.001	0.001	<0.001
	Hamilton score-phase 4	*r*			0.360	0.327	0.385
		*p*			<0.001	<0.001	<0.001
	Maternity blues score	*r*				0.086	0.123
		*p*				0.220	0.077
	Financial difficulties	*r*					0.642
		*p*					<0.001

When baseline depression score, financial difficulty score, and life stress event score, along with the dummy group variable, were entered in a regression model, financial difficulties, depression, and psychoprophylaxis sessions emerged as independent prognostic factors of maternity blues score, the group variable being most significantly associated with maternity blues (significantly low probability of maternity blues in the intervention group, *b* coefficient equal to −2.5) (Table 4).

**Table 4.** Regression Model for Maternity Blues

R^2^ = 0.308	Unstandardized coefficients	p	95.0% confidence interval for B	
	B		Lower bound	Upper bound
(Constant)	7,988	<0.001	5,845	10,131
Group	−2.453	<0.001	−2,965	−1,941
Hamilton score-phase 1	0.200	<0.001	0.088	0.312
Age	−0.017	0.466	−0.062	0.028
Financial difficulty score	0.032	0.044	0.001	0.064
Life stress events	0.001	0.448	−0.002	0.004
Dependent variable: maternity blues score				

## Discussion

According to the results of this study, the likelihood of postpartum depression and “maternity blues” was significantly reduced in the intervention group. Although financial status as well as depression continued to play a role, the deterrent contribution of psychoprophylaxis was the most important parameter in the final maternity blues prognostic model. The intervention in this study was based exclusively on a model of psychoprophylaxis involving group education and support and did not include specialized psychological approaches, while it took place prenatally; the above three characteristics constitute the originality of the study. Similar studies have been performed in the past with encouraging results; however, they did not have the three characteristics mentioned above. However, most of them advocate for the beneficial effect of the psychosomatic preparation of the pregnant woman, to reduce the frequency of postpartum depression and “maternity blues.”

Indeed, social support theory and observational studies of observational risk factors suggest that increased prenatal psychosocial support could prevent postpartum depression. A randomized controlled trial at a regional hospital clinic in China found that women who received the postpartum psychoeducation program had significantly better psychological well-being, fewer depressive symptoms, and better interpersonal relationships at 6 weeks postpartum than those who received only standard antenatal care.^19^ The intervention in that study was based on the principles of interpersonal psychotherapy and consisted of two 90-minute prenatal courses and a telephone follow-up within 2 weeks of delivery. A total of 194 women who were randomly assigned to the intervention group and the control group participated. The Edinburgh Postpartum Depression Scale, the General Health Questionnaire, and the Interpersonal Satisfaction Questionnaire were used.

The aim of the study by Collado et al^20^ was to evaluate the effect of a new psychosomatic antenatal program aimed at reducing the symptoms of depression and preterm birth. Experimental intervention using a psychosomatic approach had an impact, but did not significantly reduce the risk of postpartum depression (due to perhaps both intrinsic and exogenous factors). However, the psychosomatic approach or another interdisciplinary approach is a choice that should be considered in the antenatal care of vulnerable women in their efforts to prevent postpartum depression and preterm birth.

Both variables are important, and other studies suggest that levels of depression during pregnancy may contribute to other biomedical risk factors, such as adverse obstetric, fetal, and/or neonatal outcome.^21^ For this reason, interventions are necessary to reduce prenatal depressive symptoms. The results of our study showed potential for prevention and suggest interesting hypotheses for future interventions. In other randomized trials, the reduction in depressive symptoms in women was not significant.^13,22^

However, even in this study, the results suggest that the psychosomatic approach may be more useful to the target population than standard antenatal programs. The support women provided to one another; the exchange of ideas and the detailed information and practice about mind/body interaction through labor stages were components of the intervention and reduced postpartum depression and maternity blue in the intervention group. Even those who criticize psychoprophylaxis would hardly disagree that women interaction within a labor preparation group contributes to the between understanding of pregnancy and labor process. Regarding the high scores observed on Hamilton scale, these may be attributed to the gradual and steady increase in 1-month prevalence of major depression amid the economic downturn, along with the tendency to rise throughout pregnancy, with a steep rise in the final 8 weeks of pregnancy.

### Strengths and limitations

This study was a nonrandomized clinical trial, so it is subject to confounder restriction. This limitation was hard to avoid due to ethical consideration (we could not exclude women who would like to attend the psychoprophylaxis sessions). Selection bias could be responsible for the difference in baseline values between control and intervention group. Another limitation was that there was no control for the time spent in the intervention (and from the support from other women in the group), which may further contribute to outcome and bias the contribution of Lamaze method itself. Of note, the high rate of caesarean section in Greece is not surprising, attributed to the lack of use of international obstetric protocols and national strategies.^23^

A significant shortage of midwives. Therefore, clinical trials are required, in which random distribution of subjects results in equalizing the prognostic factors between the groups receiving different treatments. In addition, different midwives participated in this study in the provision of standard care, and there may have been differences regarding the protocols applied. Also, the women in the intervention group were about one third of the second-year students and most of them were of university level. Given this, they may be able to better manage depressive symptoms, thus increasing the effect of sessions on postpartum depression.

The quasi-experimental design of the study with the inclusion of control group, the repeated measurements, and the use of validated instruments in a primary care setting were strengths of the study, which was the first of its kind in Greece.

### Future consideration and clinical implications

This study shows the beneficial effect of psychoprophylaxis on maternal mood during pregnancy and on reducing maternity blues severity. In the era of economic crisis, which had a marked effect on mental well-being, reducing the probability of depression may be of great value. It may also improve the significance of the understanding of postpartum blues and psychoprophylaxis for childbirth. Systematic implementation of Lamaze method would lead to reduced psychiatric morbidity during pregnancy and postpartum with apparent benefits for the mother and the newborn.

Given the encouraging evidence of this study, a multicenter randomized study could be designed in the future to minimize the effect of confounding factors. A possible obstacle to conducting such a study in our country is that women are very positive about their participation in pretrial sessions when informed about them, while those who refuse to participate, do so mainly for reasons of time and distance from the center of the study. An ethical issue by the women themselves could emerge, that is in the case of randomization, that they will be deprived of an intervention that is considered essential for the outcome of the pregnancy, a reason that also acted as a deterrent to randomization in this study.

After all, the situation of women at risk due to disadvantaged socioeconomic status is worth considering, and these women may need more sessions. This is a group of women who require a more complex psychosomatic intervention during the prenatal period. Also, replicating this study with more diverse study groups, such as mothers at high risk for depression, those with multiple, complex, or multiple pregnancies, would provide further information about the effects of the program. Finally, the addition to the evaluation and clinical interview would contribute to a more accurate assessment of the severity of depression.

## Abbreviation Used

BQ: Blues Questionnaire
